# Endoscopic Evacuation Versus Open Craniotomy and Evacuation of Non-traumatic Intracerebral Bleed: A Comparative Study

**DOI:** 10.7759/cureus.62233

**Published:** 2024-06-12

**Authors:** Rajeshwari Kondabathini, Nagaraju Venishetty, Komal Usha Madineni

**Affiliations:** 1 Department of Neurological Surgery, Krishna Institute of Medical Sciences, Hyderabad, IND; 2 Department of Neurological Surgery, St. John's Medical College, Bengaluru, IND; 3 Department of Neurology, Mamata Academy of Medical Sciences, Hyderabad, IND

**Keywords:** glasgow coma scale (gcs), spontaneous intracranial hemorrhage, postoperative outcomes, neuroendoscopy, craniotomy, hematoma evacuation

## Abstract

Objective

In patients with intracerebral hemorrhage (ICH), the usage of microsurgical instrumentation and techniques can reduce traction-related injuries and enhance postoperative outcomes compared with traditional hematoma evacuation. The purpose of this study was to compare the results of endoscopic evacuation of spontaneous non-traumatic ICH with conventional open craniotomies and evacuations of ICH in terms of safety, feasibility, and neurological outcomes.

Methods

This was a prospective study that included 21 patients with spontaneous intracerebral hematomas managed by surgical evacuation endoscopically and another 24 patients with spontaneous supratentorial ICH who underwent hematoma evacuation by open craniotomy. Primary outcomes included operation duration, operative blood loss, hematoma evacuation rate, re-bleeding rate, and postoperative Glasgow Coma Scale (GCS) score.

Results

The median operation durations were 110 (90-200) and 230 (120-460) minutes in the endoscopic and open procedure groups, respectively (p = 0.00001). The median operative blood loss was 160 (80-300) and 530 (100-2000) mL in the endoscopic and open procedure groups, respectively (p < 0.00001). The median hematoma removal rates were 90% (60%-99%) and 85% (60%-100%) in the endoscopic and open procedure groups, respectively (p = 0.0348). Re-bleeding rates were higher in the endoscopic group (p = 0.46). Postoperative Glasgow Outcome Scale scores at two-month and six-month intervals were similar between the groups (p = 0.87).

Conclusion

Endoscopic hematoma evacuation for spontaneous supratentorial hemorrhage is becoming a standard surgical procedure, and promising clinical results can be expected. In addition, an endoscope can enhance time efficiency, hematoma evacuation rates, and reduce bleeding. Although endoscopic surgeries have higher re-bleeding rates, the difference is not significant when compared to open craniotomies with similar postoperative GCS scores. It is therefore important to be familiar with the endoscope and its associated equipment in order to achieve better results and reduce complications.

## Introduction

It is estimated that approximately 10-30 in 100,000 individuals develop spontaneous supratentorial intracerebral hemorrhage (ICH) annually; the mortality rate for ICH is >40% [1]. Most of the ICH survivors remain disabled. Although the clinical outcome is mainly dependent on patients’ initial clinical presentation, early surgical intervention is crucial and urgent in selected patients. Individuals with hematomas <1 cm from the cortical surface have a higher likelihood of experiencing favorable outcomes following early surgery compared with those with deep-seated hematomas [2]. However, advances in hemostatic agents and neuroendoscopy have enhanced the safety and reduced the invasiveness of the surgical removal of ICH present in deep locations. Intraoperative retraction of the brain tissue maximizes local pressure and reduces regional blood flow. These conditions may cause cerebral infarction. In addition, the retraction of cranial nerves or subarachnoid vessels may damage these structures. This surgery-related trauma may compromise postoperative outcomes. The use of microsurgical instrumentation and techniques can reduce traction-related injuries and enhance postoperative outcomes. Here, we describe the findings of our series of spontaneous supratentorial ICH patients undergoing endoscopic hematoma evacuation. Surgical indication, timing, technique, operation time, the amount of intraoperative blood loss, hematoma evacuation rate, and long-term outcomes are discussed. The aim of this study was to evaluate the safety, feasibility, and neurological outcomes following the endoscopic evacuation of spontaneous non-traumatic ICH, compared to the conventional open craniotomy and evacuation of ICH.

## Materials and methods

Study design

This is a prospective study conducted from September 2016 to October 2018 and included patients with spontaneous intracerebral hematomas treated endoscopically in the Department of Neurosurgery at Osmania General Hospital, Hyderabad. Without randomization, additional 24 patients with spontaneous supratentorial ICH who had craniotomy for hematoma evacuation were included for comparison. Informed consent was taken from the participants after the institution's ethics committee approved the study. Patients were assessed based on age, gender, clinical and radiological findings, Glasgow Coma Scale (GCS) score at presentation, during and after therapy, various management choices, and prognosis. The waiting time for operation, length of surgery time, and intraoperative blood loss were all compared. Following surgery, a CT scan was used to determine the residual hematoma. Surgical mortality and complications were observed after surgery. The Glasgow Outcome Scale (GOS) score was used to evaluate neurological results. All of the patients in the study exhibited altered sensorium, with either putaminal ICH with a hematoma volume of more than 30 ml or subcortical hemorrhage of more than 30 ml with significant mass effect (mid-line shift of more than 5 ml and sulcus effacement). Patients had to meet three inclusion criteria: putaminal or lobar ICH, volume >30 ml, and family consent. Patients with ICH caused by brain tumors, trauma, vascular lesions, or other intracranial abnormalities were excluded from the study. Patients with end-stage liver and renal disease requiring dialysis underwent surgical evacuation, if deemed necessary. Furthermore, coagulopathy was treated before the operation.

Methodology

The patients were initially assessed in the emergency care unit and stratified using the Glasgow Coma Scale. Following the initial resuscitation efforts, cranial imaging was performed. If the patient had weak respiratory effort or a low GCS score, endotracheal intubation was performed, followed by mechanical ventilation. The criteria for ventilating the patient were determined by the GCS score, hemodynamic condition, and respiratory pattern. Neurological assessments were performed once every two hours, or more frequently as needed. GCS was utilized to determine the severity of damage in all cases.

Complete blood counts, coagulation profile, serum sodium levels, serum potassium levels, fasting and post-prandial blood sugar levels, arterial blood gas analysis, renal function tests, viral studies, X-rays of the skull and cervical spine, and a CT brain scan were performed. The intracerebral hematoma volume was determined using the following formula: Clot volume = (a × b × c)/2, where a is the maximum length (in cm), b is the width, perpendicular to the length in the same CT slice, and c is the thickness of the clot, slice thickness multiplied by the number of slices. Intraventricular bleeding with dilated ventricles was also reported. CT angiography was performed in suspected cases to rule out the presence of aneurysms, arterio-venous malformations, and other vascular anomalies.

The time between the occurrence and surgery was documented. The hematoma was surgically evacuated using an endoscope. The patient and attendants thoroughly discussed the surgery technique, as well as any intraoperative and postoperative complications. The coagulopathy state was addressed prior to the procedure. Patients with low platelet counts had platelet infusion, whereas patients with an abnormal international normalized ratio (INR) received fresh frozen plasma (FFP) and vitamin K. Platelet infusion was required for patients using antiplatelet drugs (such as aspirin or clopidogrel), despite having normal coagulation parameters and platelet counts. The bleeding site served as the point of the skull entry. Frazier's point, Kocher's point, Keen's point, which are comparable to ventriculostomy entry locations, were used based on the site of bleeding. It was only the trajectory that varied. As a result, Kocher's site was the ideal entry point, with a similar lateral trajectory as a ventriculostomy if the patient had putamen ICH. It might have been a better option to use Keen's point of entry in the case of thalamic ICH with temporal extension. For an occipital extension of the ICH, Frazier's point was perfect. The intracranial pressure (ICP) of all patients with ICH was monitored with a contralateral ventriculostomy.

Anaesthesia was administered, and then a linear scalp incision of approximately 4.5-5 cm was made. We then drilled a 4-cm width burr hole. The dura was then opened in a U shape after tenting. The ventricular catheter was placed through a sutured glove through a 1-cm cerebral incision created by bipolar cauterization. A tract was formed by inflating the glove. This was followed by the application of a translucent plastic sheath. As soon as a channel was created, an endoscope was inserted into the deeper part of the hematoma. Pressure increases in hematomas caused them to ooze. Transparent sheaths provide excellent field of visual impression, so the innermost part of the hematoma was evacuated initially, followed by progressive withdrawal of the sheath, pushing the residual hematoma towards its tip. A zero-degree rod-lens endoscope, angle-dependent suction (anterior or posterior with a diameter of 5, 7, or 10 mm), and rotation of the sheath are excellent tools to remove hematomas in extreme angles. Suction adapters allow for quick changes in the suction power. If there is no active bleeding, hemostatic drugs are used to control seeping. A surgeon used bipolar forceps to cauterize aggressive bleeders. Both the operation time and blood loss were recorded.

The systolic blood pressure was tightly regulated with a target of less than 160 mm Hg after surgery. Additional fluids were avoided to prevent volume overload and resultant hypertension. A ventricular catheter was used to monitor the ICP, which was kept below 20 mm of water. A brain CT scan was performed as soon as a patient was stabilized following hematoma evacuation. The volume of each hematoma was calculated by adding the contoured regions on each slice and multiplying with the slice thickness (approximately 5 mm). Using the trapezoidal rule, with proper demarkation on a minimum of five slices, a 10% error rate was expected for tumor volume estimation in radiosurgery. Thus, when used for CT imaging of a three-dimensional target such as a hematoma, this type of measurement has a 10%-20% uncertainty [3]. In order to calculate the hematoma evacuation rate, preoperative hematoma volume was subtracted from postoperative hematoma volume. At two and six months following the operation, we obtained GOS scores to document any morbidity and death.

Statistical analysis

The descriptive statistics for continuous variables were medians and ranges, while the descriptive statistics for categorical variables were frequencies and percentages. Analysis was conducted using parametric tests since the study variables were normally distributed. For categorical variables, cross-tabulations were created, and Fisher's exact, Pearson's chi-square or Mantel-Haenszel tests were used to compare distributions. In order to assess whether continuous variables had equal variances between subsets of patients classified by categorical data, independent t-tests were conducted. The statistical significance was set at p < 0.05.

## Results

A total of 45 patients were admitted to the Department of Neurosurgery at Osmania General Hospital. Patients underwent a variety of surgical techniques, including endoscopic evacuation of intracerebral hematoma, open craniotomy, hematoma evacuation, external ventricular drainage, and ventriculoperitoneal shunts. Table 1 shows demographic information on the study population, which consisted of 45 admitted cases.

**Table 1:** Demographics and preoperative characteristics of the study population N, number; GCS, Glasgow Coma Scale; ICH, intracranial hemorrhage; IVH, intraventricular hemorrhage

No.	Variables	Sub-categories	N (%)
1	Age group distribution (years)	<40	2 (4.5%)
		41-50	6 (13.3%)
		51-60	23 (51%)
		61-70	10(22.2%)
		>70	4 (8.8%)
2	Sex	Male	34 (75.5%)
		Female	11 (24.5%)
3	Clinical presentation	Headache	30 (66%)
		Hemiparesis	28 (62%)
		Altered sensorium	25 (55%)
		Vomiting	18 (40%)
		Aphasia	12 (26%)
4	Time of presentation	6-24 hours	15 (33.3%)
		After 24 hours	30 (66.6%)
5	GCS score at presentation	3-8	7 (15%)
		9-12	28 (62%)
		13-15	10 (22%)
6	Location of ICH	Putamen	33 (73%)
		Lobar (sub-cortical)	12 (27%)
7	ICH with IVH	Present	30 (66.7%)
		Absent	15 (33.3%)
8	Comorbidities	Hypertension	41 (91%)
		Diabetes mellitus	26 (57%)
		Coagulopathy	2 (4.4%)
		Liver cirrhosis	1 (2.2%)
		Renal insufficiency	1 (2.2%)
9	Volume of ICH	30-50 ml	14 (31%)
		50-80 ml	28 (62%)
		>80 ml	3 (6%)

All cases were divided into age groups, with the age ranging from under 40 years to over 70 years. The most common age group of presentation was 51-60 years, accounting for 51% of the study population, whereas the mean age of presentation was 50. There were 34 male and 11 female patients, giving a male-to-female ratio of 3:1. There was a slight male predominance in the current study. Patients exhibited a variety of clinical characteristics as described in the study. The most prevalent form of presentation was headache in 30 (66%) individuals, followed by hemiparesis in 28 (62%), and altered sensorium in 25 (55%) patients. Additional forms of presentation included vomiting, aphasia, and cranial nerve palsies. The total number of patients who presented to the department following the start of the first intolerable symptom and the symptom detected by the attendees was 15 (33.3%), who presented at 6 to 24 hours, and 30 patients (66.6%), who presented after 24 hours.

Patients who present to the department were grouped based on their GCS score at the time of presentation. There were 28 patients, or 62% of the cases, with a GCS score of 9-12 at presentation. Patients with a GCS score of 13-15 and 3-8 were 10 (22%) and 7 (15%), respectively. Of the patients, 33 patients (73% of the cases) had a hematoma in the putamen, while 12 patients (27% of the cases) had a hematoma in the lobar (subcortical) area. Of the total number of patients included in the study, 30 patients (66.7%) had an intraventricular hemorrhage, while the remaining patients had no intraventricular extension. The study comprised 41 (91%) patients with hypertension, 26 (57%) individuals with diabetes mellitus, and a few others with co-morbidities such as coagulopathy, liver cirrhosis, and renal insufficiency. The patients were divided into three groups based on the volume of the hematoma as assessed by the CT scan. A total of 28 (62%) patients had a volume ranging from 50 to 80 ml, 14 (31%) had a volume between 30 and 50 ml, and 3 (6%) had a volume greater than 80 ml. Of the total number of patients included in the study, 21 (46%) received endoscopic evacuation of the spontaneous intracerebral hematoma, whereas 24 (54%) underwent open craniotomy and evacuation.

**Figure 1:**
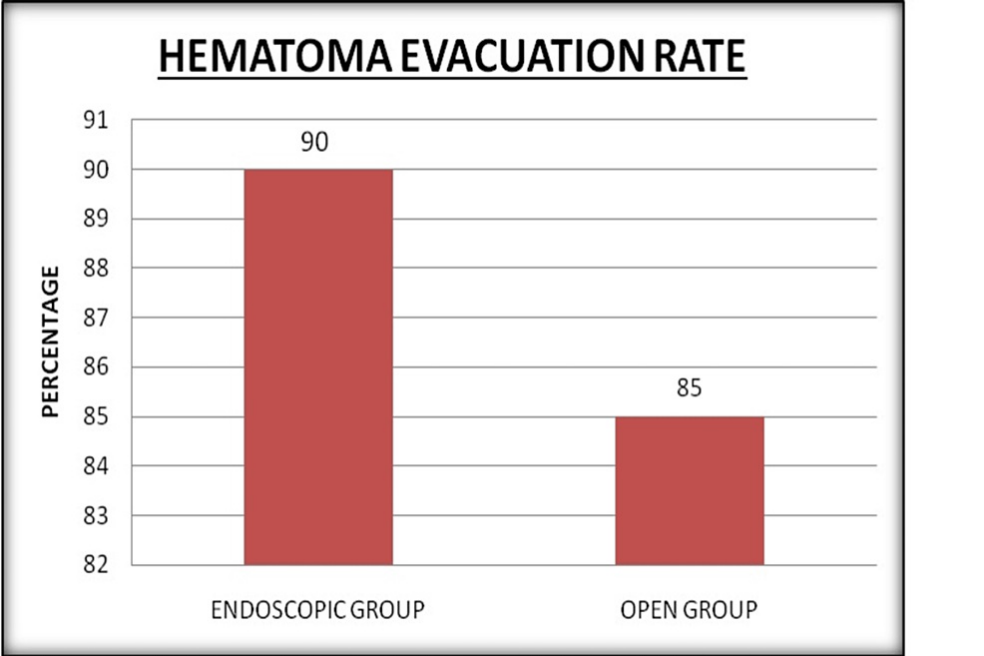
A comparative analysis of hematoma evacuation rates (in percentages) between the endoscopic group and open craniotomy group in patients with spontaneous intracerebral hemorrhage

**Figure 2:**
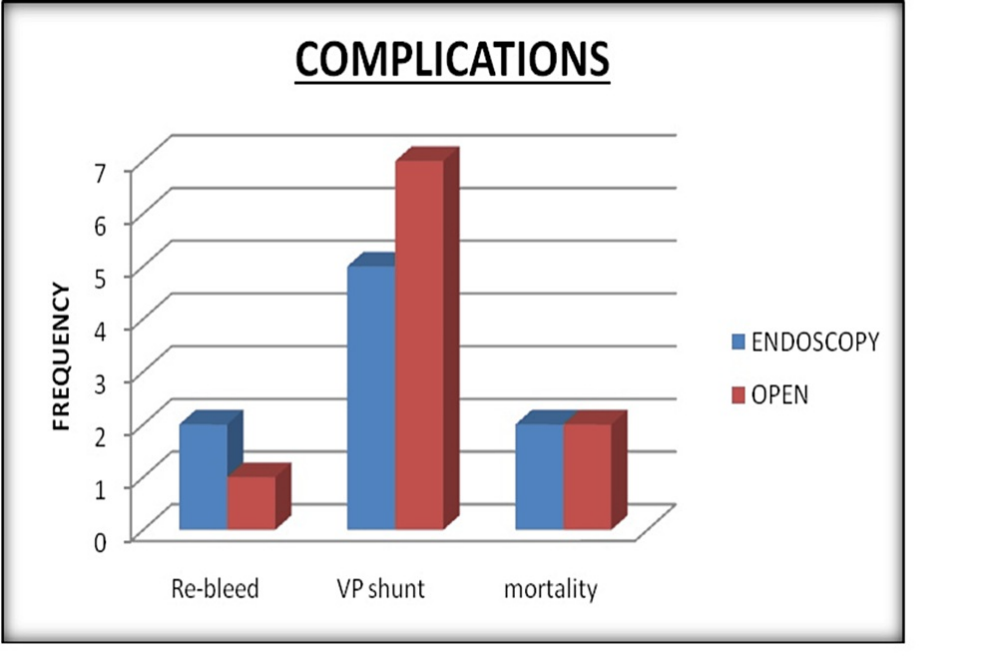
A comparative analysis of postoperative complications (in frequencies) between the endoscopic group and open craniotomy group in patients with spontaneous intracerebral hemorrhage VP shunt, ventriculoperitoneal shunt

The median time for endoscopic hematoma evacuation was 110 minutes, with a range of 90-200 minutes, while the median time for open craniotomy hematoma evacuation was 230 minutes, with a range of 120-460 minutes. An endoscopic procedure resulted in a median blood loss of 160 ml (80-300 ml). An open surgery resulted in a median blood loss of 530 ml (100-2000 ml). The median hematoma evacuation rate in the endoscopic group was 90%, with a range of 60%-99%. The open group had a median hematoma evacuation rate of 85%, ranging from 52% to 100%. Two (9.5%) patients in the endoscopic group re-bled, as did one (4%) in the open group. Five patients (23%) in the endoscopic group required a permanent ventriculoperitoneal shunt, while 7 (28%) in the open group did. Two (9.5%) patients in the endoscopic group died, while 2 (8%) patients in the open group died (Figure 1). The median postoperative GCS score in the endoscopic group was 11, with a range of 8 to 15, while in the open group, it was 11, with a range of 4 to 15. The median GOS score in the endoscopic group was 3 (1-4) at two and six months after surgery, respectively. The median GOS score in the open group was 3 (1-4) at two and six months after surgery, respectively (Figure 2). Table 2 provides details about the postoperative characteristics of the study participants. Table 3 provides details about the postoperative complications and outcome scores of the study participants.

**Table 2:** Comparison of the postoperative characteristics among sub-categories of patients with intracranial hemorrhage GCS, Glasgow Coma Scale; VP shunt, ventriculoperitoneal shunt; n, number of patients in each sub-category

No.	Variables	Sub-categories (n)	Postoperative findings	p-value
1	Median operation time (minutes)	Endoscopy group (21)	110 minutes (90-200 min)	< .00001
		Craniotomy group (24)	230 minutes (120-460 min)	
2	Median blood loss (ml)	Endoscopy group (21)	160 ml (80-300 ml)	< .00001
		Craniotomy group (24)	530 ml (100-2000 ml)	
3	Median hematoma evacuation rate (%)	Endoscopy group (21)	90% (60%-99%)	0.0348
		Craniotomy group (24)	85% (52%-100%)	
4	Median GCS score post-procedure	Endoscopy group (21)	11 (8-15)	0.0898
		Craniotomy group (24)	11 (4-15)	

**Table 3:** Comparison of postoperative complications and outcome scores of the study participants among various sub-categories GCS, Glasgow Coma Scale; GOS, Glasgow Outcome Scale; VP shunt, ventriculoperitoneal shunt; n, number of patients

No.	Variables	Sub-categories		p-value
1	Postoperative complications in each sub-category	Endoscopy group (n = 9) (%)	Craniotomy group (n = 10) (%)	
	Re-bleed	2 (9.5%)	1 (4%)	0.46
	VP shunt	5 (23%)	7 (28%)	0.42
	Mortality	2 (9.5%)	2 (8%)	0.90
2	GOS score (GOS)	Endoscopy group (19)	Craniotomy group (22)	
	Mean GOS score at 2-month follow-up	3 (1-4)	3 (1-4)	0.87
	Mean GOS score at 6-month follow-up	3 (1-4)	3 (1-4)	0.87

## Discussion

Mechanical compression and the neurotoxic effect of hematomas have been linked to brain damage during ICH [4]. Early surgery to minimize mechanical compression of the normal brain tissue and neurotoxic consequences of hematoma may limit brain harm, but whether and when to do surgical ICH evacuation is still debatable [5]. The excision of supratentorial ICH with routine craniotomies may be considered for patients with lobar ICH greater than 30 ml and within 1 cm of the cortical surface [6]. However, minimally invasive ICH evacuation with endoscopic methods is still under evaluation [6]. As the neuroendoscopic system and instruments have advanced, current findings show that the rate of ICH evacuation has increased to 99% [2].

Patients in this study ranged in age from under 40 to over 70 years. The most typical age group for the presentation was 51 to 60 years. ICH was more common in men. These findings align with those reported by Xu et al. in 2018 [7]. They found that ICH was more common in people aged 66 years, with a mean age of 40 to 70 years, and in men, with a male-to-female ratio of 3:1.

In our study, headaches were the most common manifestation in 30 (66%) individuals, followed by hemiparesis in 28 (62%) and altered sensorium in 25 (55%) patients. The clinical appearance and subsequent course vary between instances. It is understood that consciousness levels deteriorate in an unanticipated and quick manner. Most patients whose awareness levels gradually fall following presentation experience this deterioration within 72 hours of symptom start. Hyperacute presentation is associated with poor results. Approximately one-third of patients are comatose upon admission. With the broad availability of imaging, it has become increasingly clear that some individuals with intracerebral hematoma have a more benign course.

In our study, 28 (62%), 10 (22%), and 7 (15%) patients received a GCS score from 9 to 12, 13 to 15, and 3 to 8, respectively. Given the extensive availability of imaging modalities, the majority of patients had an acceptable GCS score at presentation. GCS scores have a strong correlation with outcomes and can predict surgical results. Surgical death rates rise with lower preoperative levels of awareness.

Hematoma volume at admission is an important determinant in influencing mortality and functional outcomes. CT allows for the precise measurement and evaluation of hematoma dimensions during a clinical examination. In our study, 28 (62%), 14 (31%), and 3 (6%) patients had hematoma volumes of 50-80 ml, 30-50 ml, and more than 80 ml, respectively. Furthermore, at presentation, 33 (73%) and 12 (27%) individuals exhibited bleeding in the putamen and lobar (subcortical) regions, respectively. Broderick and colleagues found that ICH volume was the best predictor of 30-day death, regardless of the hematoma site. Patients with ≥60-ml hematomas and GCS scores ≤7 had a 91% projected death rate within 30 days. Only one of 71 patients with ICH more than 30 ml attained functional independence after 30 days. There was no significant difference in outcomes between surgical and medicinal therapy arms for patients with hematomas of less than 30 ml. Among four patients expired in this study, one had an ICH volume of 72 ml, and the other three had a volume of 84 ml, 82 ml and 92 ml each, respectively. Qureshi and colleagues found that the death rate increased dramatically when the hematoma volume exceeded 30 ml [8]. Re-hemorrhage, which typically happens within the first six hours after the first bleed, is another sign of a bad prognosis [9]. Lower GCS scores and medical comorbidities all contribute to a negative prognosis.

In a controlled randomized trial, Auer et al. compared endoscopic evacuation with medical treatment in 100 patients with spontaneous supratentorial intracerebral hematomas (subcortical, putaminal, and thalamic) [10]. Patients with subcortical hematoma who underwent surgery had considerably decreased mortality rates compared to those who received medical care (30% vs. 70%, p < 0.05). Furthermore, 40% reported satisfactory results with little or moderate shortfall. Patients with smaller than 50 cm^3 ^hematomas who underwent surgery had considerably better functional recovery than those who received therapy medically.

Nakano et al. compared neuroendoscopic surgery to standard treatment for ICH [11]. They identified the following indications for endoscopy: deep-seated hematomas (e.g., thalamic and intraventricular hematomas), putaminal small- to medium-sized hematomas, and inability to tolerate general anesthesia. In this study, 21 (46%) patients had a spontaneous intracerebral hematoma removed along with a contralateral external ventricular drain installation, while 24 (54%) had an open craniotomy with hematoma removal. The endoscopic surgery and open procedure groups had median operation durations of 110 (90-200) and 230 (120-460) minutes, respectively (p < 0.00001). Surgical blood loss was 160 (80-300) ml in the endoscopic procedure group and 530 (100-2000) ml in the open procedure group (p < 0.00001). Hematoma clearance rates were 90% in the endoscopic surgery group and 85% in the open surgery group (p = 0.034).

Cho et al. conducted a prospective study to assess the neurological results, safety, and cost-effectiveness of three surgical treatments for spontaneous basal ganglia bleeding [12]. They randomly assigned 90 non-comatose patients to three groups. It was found that the craniotomy group had a longer operative time of 229.96 minutes and the maximum blood loss (236.13 ± 137.45 ml; p < 0.001). The endoscopic group achieved the highest hematoma clearance rate (87% ± 8%; p < 0.01). The endoscopic group showed greater improvement in muscle power in afflicted limbs compared to the craniotomy group (p = 0.004). Compared to craniotomies, endoscopic surgery was more cost-effective. These findings are consistent with those of our investigation.

In a retrospective research, Xu et al. compared the effectiveness of endoscopic surgery against craniotomy for supratentorial hypertensive ICH [7]. Endoscopy and craniotomy were done on 82 and 69 individuals, respectively. Hematoma removal rates were 90.5% ± 6.5% and 82.3% ± 8.6% with endoscopy and craniotomy, respectively (p < 0.01). The endoscopy and craniotomy groups had operating durations of 1.6 ± 0.7 and 5.2 ± 1.8 hours, respectively (p < 0.01). Blood loss during surgery was 91.4 ± 93.1 ml and 605.6 ± 602.3 mL for endoscopy and craniotomy, respectively (p < 0.01). These findings are consistent with those of this investigation.

Re-bleeding occurred in 2 (9.5%) in the endoscopic group and 1 (4%) in the open group (p = 0.591). Furthermore, 5 (23%) and 7 (28%) patients in the endoscopic and open groups, respectively, needed a permanent ventriculoperitoneal shunt (p = 0.746). Two (9.5%) in the endoscopic group and 2 (8%) patients in the open group died after surgery (p = 0.591). In the aforementioned investigations, the endoscopy group had lower rates of re-bleeding, death, and morbidity than the craniotomy group due to less nearby tissue injury and blood loss, as well as shorter surgical time. Minimally invasive surgery has a small wound size and causes little brain tissue stress. The establishment of a good working channel is critical in endoscopic surgery. Several working channels have been developed, including transparent and handcrafted sheaths. However, the tract must be built in a safe and simple manner. We used a homemade rubber balloon catheter to produce the tract, followed by a retractor or transparent sheath. Hirsch et al. used a rubber balloon catheter to treat profound intracerebral lesions for the first time in 1990 [13]. This method can help to decrease nervous tissue damage along the course [14]. Although we observed ICP increase during balloon inflation, the amount of the elevation was reduced because we drained contralateral extraventricular cerebrospinal fluid. The inflating time was less than four minutes, which is the maximum period allowed for cerebral ischemia. Chen et al. created a polypropylene sheath to improve endoscopic vision and thalamic hematoma evacuation efficiency [15].

Endoscopic surgery presents a challenge in maintaining hemostasis. Managing hemostasis in endoscopic surgery is more challenging than in microsurgery due to surgeons' unfamiliarity with the freehand endoscopic approach and tool constraints. Although multifunctional instruments that address these restrictions are being developed, the expensive cost of equipment remains a concern. In our facility, we only use suction for hematoma evacuation and bipolar forceps to coagulate leaking tiny capillaries. Compared to entire hematoma evacuation, fast decompression is more important. Thus, we use suction to choose the hematoma location for removal and still see a tiny hematoma on the surrounding brain tissue.

In the case of modest oozing, we employ hemostatic drugs (such as Floseal) rather than coagulants. Our re-bleeding rate of 9.5% (2 of 21 patients) is greater than in previous research [16]. Due to poor hypertension control, these two patients experienced re-bleeding three days after surgery rather than shortly afterward. Thus, strict blood pressure control following surgery can help prevent re-bleeding and hemostasis.

The majority of our patients had improved GCS scores one week after surgery. Two individuals had low GCS scores as a result of recurrent bleeding. For functional recovery, the median GOS score was 3 at two and six months after surgery. These findings are consistent with other studies that found increased functional recovery postoperatively in the short term but not in the long term [17].

Endoscopic spontaneous ICH removal is only appropriate for certain patients (those with GCS scores more than 8 and modest ICH volume) [6]. However, no study has shown an improvement in minimally invasive endoscopic outcomes. American Heart Association/American Stroke Association guidelines do not support early supratentorial ICH removal as a way to improve functional outcomes. There is a higher risk of recurrent bleeding with early craniotomies. However, Kuo et al. showed that early endoscopic evacuation (within 12 hours) resulted in a high evacuation rate [16].

The study was limited by a small sample size. While we performed early surgery on ICH patients requiring surgical bleed evacuation, we were not able to properly establish the proper period of time within which the prognosis is better. We performed endoscopic surgery on 47% (n = 21/45) of patients who received operative intracranial bleed evacuation. Surgical procedures were not performed on patients with poor prognostic factors like GCS score >3, unstable hemodynamic status, and uncontrolled coagulation parameters. As a result, the findings may be more positive than those of the overall population. The effectiveness of surgical hematoma removal in patients with preoperative GCS scores <8 is still debated, but some individuals have shown satisfactory recovery. Endoscopic spontaneous supratentorial hematoma excision is becoming a common surgical treatment, with encouraging results. An endoscope can provide a clear vision, speed up the treatment, and prevent hemorrhage. As a result, surgeons should understand endoscopy and accompanying instrumentation in order to achieve positive outcomes with few problems. An endoscope is relatively expensive and may not be available in resource-poor settings. The endoscopic view is two-dimensional compared with the three-dimensional view provided by microsurgical techniques. To validate our findings, large cohorts and long-term follow-ups are needed, particularly to compare open craniotomy with neuroendoscopic surgery and refine the optimal surgical approach for spontaneous non-traumatic ICH.

## Conclusions

In conclusion, our study supports endoscopic hematoma evacuation as a viable and effective alternative to traditional open craniotomy for spontaneous supratentorial intracerebral hemorrhage. The endoscopic approach demonstrated advantages such as shorter operation durations, reduced operative blood loss, and comparable hematoma removal rates. While re-bleeding rates were slightly higher in the endoscopic group, mortality rates were comparable between the two approaches. The promising clinical results observed in endoscopic hematoma evacuation underscore its role as an evolving and effective surgical modality. Continued research and advancements in neuroendoscopy are essential to refine techniques, improve patient selection criteria, and further enhance the overall outcomes for individuals with spontaneous intracerebral hemorrhage.
